# Lipid metabolism in autoimmune rheumatic disease: implications for modern and conventional therapies

**DOI:** 10.1172/JCI148552

**Published:** 2022-01-18

**Authors:** George Robinson, Ines Pineda-Torra, Coziana Ciurtin, Elizabeth C. Jury

**Affiliations:** 1 Centre for Rheumatology Research,; 2 Centre for Adolescent Rheumatology Research, and; 3 Centre for Cardiometabolic and Vascular Science, Division of Medicine, University College London, London, United Kingdom.

## Abstract

Suppressing inflammation has been the primary focus of therapies in autoimmune rheumatic diseases (AIRDs), including rheumatoid arthritis and systemic lupus erythematosus. However, conventional therapies with low target specificity can have effects on cell metabolism that are less predictable. A key example is lipid metabolism; current therapies can improve or exacerbate dyslipidemia. Many conventional drugs also require in vivo metabolism for their conversion into therapeutically beneficial products; however, drug metabolism often involves the additional formation of toxic by-products, and rates of drug metabolism can be heterogeneous between patients. New therapeutic technologies and research have highlighted alternative metabolic pathways that can be more specifically targeted to reduce inflammation but also to prevent undesirable off-target metabolic consequences of conventional antiinflammatory therapies. This Review highlights the role of lipid metabolism in inflammation and in the mechanisms of action of AIRD therapeutics. Opportunities for cotherapies targeting lipid metabolism that could reduce immunometabolic complications and potential increased cardiovascular disease risk in patients with AIRDs are discussed.

## Introduction

The inflammation associated with autoimmune rheumatic diseases (AIRDs), including rheumatoid arthritis (RA) and systemic lupus erythematosus (SLE), is dependent on multiple immune cell subsets within disease-specific settings, each having different metabolic demands (1). For example, effector T cells are dependent on glycolytic metabolism for their growth and effector functions, whereas regulatory T cells utilize lipids via mitochondrial β-oxidation and the generation of ATP through oxidative phosphorylation (OXPHOS) (2). Naive B cells are maintained in a reduced metabolic state, while their activation relies on metabolic programming toward OXPHOS (3). Similarly, during inflammation, inflammatory M1 macrophages use glycolysis, whereas more antiinflammatory M2 macrophages typically use β-oxidation (4). Autoinflammatory responses in AIRDs have high energy demands and involve elevated lipogenesis, glucose and glutamine metabolism, and a switch toward cellular glycolysis from OXPHOS for energy metabolism. For example, hypoxia in the RA synovium induces chronic T cell mitochondrial hyperpolarization associated with increased glucose metabolism and ATP synthesis, and in SLE patients and lupus-prone mice, chronically activated T cells have increased mitochondrial glucose oxidation and hyperpolarization, as reviewed previously (1, 5). Many of the current therapies for AIRDs influence lipid metabolic pathways to exert their therapeutic benefit; furthermore, lipid metabolism could play a role in the pathogenesis of AIRDs and associated comorbidities. This Review discusses lipid metabolism in inflammation and mechanisms of action of AIRD therapeutics, addresses the on- and off-target metabolic effects induced by these therapies, and highlights opportunities for cotherapies targeting lipid metabolism that could benefit patients, with a focus on cardiovascular disease (CVD) risk, an important comorbidity in patients with AIRDs.

## Lipid metabolic pathways in inflammation

**Figure 1:**
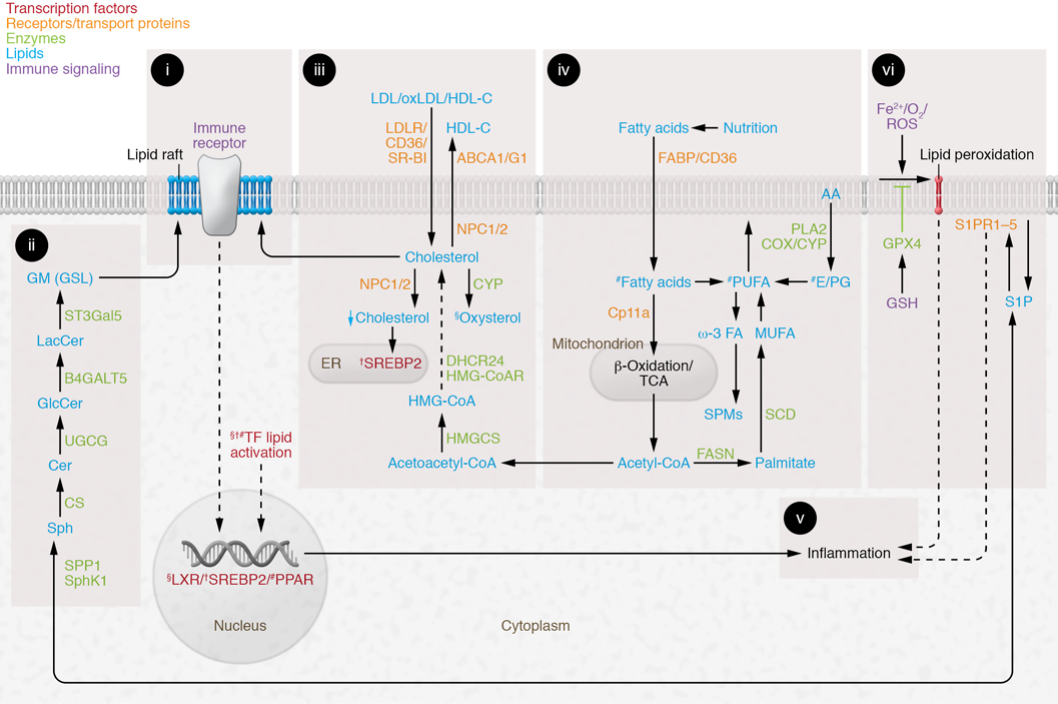
Summary of lipid metabolism pathways important for inflammation. (i) Lipid rafts: cholesterol- and glycosphingolipid (GSL)-enriched cell signaling platforms. (ii) De novo GSL biosynthesis: differential expression influences immune receptor–mediated signaling and cell function. (iii) Intracellular cholesterol is regulated by liver X receptor (LXR) and sterol regulatory element–binding protein 2 (SREBP-2) (see v). LXR activation by oxysterols induces cholesterol efflux (ABCA1, ABCG1) and reduces lipid uptake (LDLR, VLDLR, SR-BI, CD36). Niemann-Pick type C1 (NPC1) and NPC2 regulate lysosomal/late endosomal trafficking/recycling of intracellular lipids. SREBP-2 opposes LXR and promotes cholesterol biosynthesis and uptake (*HMGCoAR*, *LDLR*). (iv) Nutrition influences fatty acid composition and metabolism. Fatty acids are metabolized to produce energy (ATP) by mitochondrial β-oxidation and TCA. Monounsaturated fatty acids (MUFAs) are synthesized via acetyl-CoA, fatty acid synthase (FASN), and stearoyl-CoA desaturase (SCD). Polyunsaturated fatty acids (PUFAs), diet-derived or biosynthesized in vivo, influence arachidonic acid (AA) metabolism. PUFAs are precursors to triglycerides, phospholipids in plasma membrane, second messengers, hormones, and ketone bodies. Prostaglandins (PGs) are produced following AA release from membrane phospholipids. Downstream PG signaling and eicosanoids have direct metabolic effects on immune cells via PPARs, mediating antiinflammatory effects and modulating LXRs (see v). Omega-3 (ω-3) PUFAs are enzymatically converted to antiinflammatory resolvins (specialized pro-resolving mediators [SPMs]). (v) Lipid metabolism activates transcription factors and influences inflammation via multiple mechanisms (see text). (vi) PUFA phospholipid peroxidation is induced by iron overload and ROS. Products of lipid peroxidation are eliminated via glutathione peroxidase-4 (GPX4); clearance defects induce cell membrane damage and ferroptosis. Sphingosine 1-phosphate (S1P) is derived from membrane phospholipids; activation via binding to S1P receptors (S1PR1–5) initiates immune cell localization to inflammatory sites and T cell differentiation. §, †, and # indicate processes activating LXR, SREBP2, or PPARs, respectively. Other abbreviations: B4GALT5, β-1,4-galactosyltransferase-5; Cer, ceramide; CS, ceramide synthase; DHCR24, 24-dehydrocholesterol reductase; FABPs, fatty acid–binding proteins; GlcCer, glucosylceramide; GM, GM1 ganglioside; GSH, glutathione; HMGCS, 3-hydroxy-3-methylglutaryl–CoA synthase-1; LacCer, lactosylceramide; ox, oxidized; Sph, sphingosine; SphK1, sphingosine kinase-1; SPP1, S1P phosphohydrolase-1; SR-BI, scavenger receptor class B type I; ST3Gal5, ST3 β-galactoside α-2,3-sialyltransferase-5; UGCG, UDP-glucose ceramide glucosyltransferase.

Lipid metabolism is a crucial aspect of cellular metabolism and effective immune responses; lipids derived from both de novo biosynthesis and exogenous sources are essential for fuel and growth and as membrane components, and mediate cell signaling (6), as summarized in Figure 1. Aspects of lipid metabolism that could contribute to disease pathogenesis and associated comorbidities in patients with AIRDs and serve as therapeutic targets are outlined below.

### Immune cell lipid membranes and dyslipidemia

Immune cell plasma membranes comprise a phospholipid bilayer containing highly ordered areas enriched in glycosphingolipids and cholesterol, called lipid rafts. Immune cell surface receptors (including T cell and B cell receptors and costimulatory molecules) reside within lipid rafts and facilitate appropriate cell signaling in response to antigen or other cellular ligands (refs. 7–9 and Figure 1, A–C). Lipid rafts are altered in SLE, in which an increase in both cell membrane glycosphingolipids and cholesterol attributable to increased cellular lipid synthesis is associated with increased T cell and B cell receptor signaling and ultimately activation and inflammation (10, 11). Circulating lipids also play a role in immune cell function, through lipid uptake from VLDL or LDL via the VLDL or LDL receptor (VLDLR or LDLR), respectively, or from HDL via scavenger receptor class B type I (SR-BI) and CD36; or through lipid efflux to HDL or apolipoprotein A1 via ATP-binding cassette transporter A1 (ABCA1) and ABCG1. Both uptake and efflux influence cellular lipid burden and function (ref. 12 and Figure 1C). This is of particular importance in autoimmunity, where dyslipidemia and cardiovascular complications are common (13). Many studies report altered lipoprotein metabolism in AIRDs. This includes reduced serum total cholesterol, triglycerides, and LDL-cholesterol (LDL-C) in patients with untreated RA that increase when patients are treated (the so-called lipid paradox in RA; ref. 14), and elevated LDL-C and medium-chain/free fatty acids and reduced HDL and long-chain fatty acids in SLE (15–18). Interestingly, a dysfunctional proinflammatory HDL, lacking the antioxidant capacity of conventionally cardioprotective HDL, is described in 48.2% of women with SLE (15), and in patients with active RA (19), highlighting the prevalence of this form of HDL in AIRDs that is rarely measured and could be associated with elevated cardiovascular risk.

### Pro- and antiinflammatory lipid metabolism

Proinflammatory lipids including eicosanoids contribute to some of the typical clinical symptoms associated with many AIRDs, such as joint pain, stiffness, and swelling (ref. 20 and Figure 1D). Eicosanoids are produced following the phospholipase A_2_–mediated release of arachidonic acid from membrane phospholipids (21). Arachidonic acid (an essential polyunsaturated omega-6 fatty acid) is subsequently converted into active metabolites by cyclooxygenase (COX), lipoxygenase, and cytochrome p450 (CYP) enzymes (20, 22). Downstream eicosanoid signaling can have a direct metabolic effect on immune cell subsets via the modulation of PPARs, nuclear hormone receptors that mediate antiinflammatory effects and modulate liver X receptors (LXRs), regulators of cholesterol homeostasis (23). Prostaglandin signaling can either stimulate (prostaglandin D_2_) or inhibit (prostaglandins F_2α_ and E_2_) the antiinflammatory ability of PPARγ to antagonize NF-κB in multiple immune cells, including T cells, B cells, macrophages, and dendritic cells (refs. 24, 25, and Figure 1E). PPARγ has been detected in macrophage-rich regions of human atherosclerotic plaques, where it controls lipid homeostasis and inhibits activation induced by proinflammatory cytokines (26). Although the functions of PPARs are tissue specific, some functions affecting lipoprotein metabolism include induction of lipolysis, reduced cellular triglyceride synthesis and VLDL production, enhanced intracellular LDL catabolism, and increased HDL production (27).

More recently, lipid metabolites termed specialized pro-resolving mediators (SPMs) have been shown to mediate resolution of inflammation and the restoration of tissue homeostasis. SPMs (including lipoxins, resolvins, and protectins) are produced by immune cells from the enzymatic conversion of omega-3 fatty acids and could be dysregulated in the context of AIRDs (refs. 20, 28, and Figure 1D). SPM levels correlate with decreased joint pain in RA patients (29) and are reduced in experimental models of RA with non-resolving joint inflammation (30). SPMs and other metabolites could also serve as biomarkers for predicting drug efficacy. A machine learning study in RA identified that peripheral blood SPM levels correlated positively with antirheumatic drug responsiveness at 6 months; notably decreased SPM concentrations were seen in nonresponders (31).

### Sphingosine 1-phosphate

Sphingosine 1-phosphate (S1P), derived from membrane phospholipids, exerts its effect by binding GPCRs (S1P receptors) (Figure 1F). S1P binding initiates multiple cellular and physiological events, including immune cell localization to inflammatory sites, trafficking of lymphocytes to and from secondary lymphoid organs, and the regulation of T cell differentiation (between proinflammatory Th17 cells and Tregs) (32). Interestingly, HDL is an important S1P chaperone, and cellular uptake of HDL-S1P by SR-BI facilitates binding to S1P receptors with subsequent signaling in a cell-specific manner (33). Targeting of S1P receptors is an effective therapy to inhibit lymphocyte trafficking in patients with multiple sclerosis, and clinical trials are exploring similar therapies in AIRDs (34).

### Ferroptosis

Ferroptosis is a form of regulated cell death driven by iron-dependent lipid oxidation (Figure 1F). Peroxidation of phospholipids containing polyunsaturated fatty acids is mediated by iron overload (free iron and iron-dependent lipoxygenases) and ROS. Under normal homeostatic conditions, cells can eliminate products of lipid peroxidation via several complex mechanisms (including glutathione peroxidase 4, an inhibitor of phospholipid peroxidation); however, in numerous conditions, including inflammation, these mechanisms are defective, leading to cell membrane damage and ferroptosis (35). Notably, increased ROS levels induced during inflammation in AIRDs could contribute to increased ferroptosis (36). Higher concentrations of free iron and other iron-binding proteins in synovial fluid and infusion of iron-dextran increase lipid peroxidation, decrease red cell glutathione, and exacerbate RA synovitis (36). In SLE, increased dietary iron and iron infusion also exacerbate disease activity (37). However, it remains to be confirmed whether inhibiting ferroptosis could be beneficial in AIRDs (36, 37).

## Conventional antiinflammatory therapies

**Figure 2:**
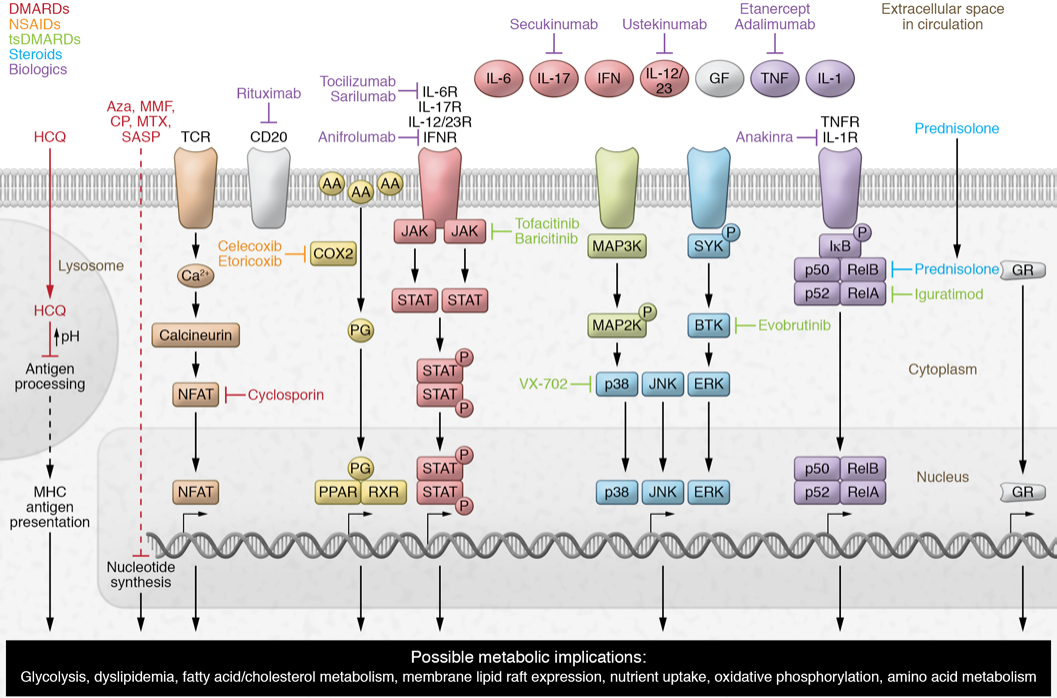
Summary of the mechanisms of action of current therapies used in AIRDs. Schematic representation summarizing the key mechanistic pathways affected by both traditional and modern therapies used to treat AIRDs, including disease-modifying antirheumatic drugs (DMARDs), target synthetic DMARDs (tsDMARDs), nonsteroidal antiinflammatory drugs (NSAIDs), steroids, and biologics. The majority of these therapeutics result in the modification of immune functions and metabolic pathways through alterations in gene transcription. These pathways provide insight into opportunities for cotherapies to prevent off-target immunometabolic effects. AA, arachidonic acid; Aza, azathioprine; CP, cyclophosphamide; GF, growth factor; GR, glucocorticoid receptor; HCQ, hydroxychloroquine; NF-κB, nuclear factor NF-κB (p50/p52/RelA/RelB/); IκB, inhibitor of κB; MAP2/3K, mitogen-activated protein 2-kinase or 3-kinase; MMF, mycophenolate mofetil; MTX, methotrexate; NFAT, nuclear factor of activated T cells; PG, prostaglandin; R, receptor; RXR, retinoid X receptor; SASP, sulfasalazine; SYK, spleen-associated tyrosine kinase; TCR, T cell receptor.

Many of the antiinflammatory therapies used to treat AIRDs influence multiple metabolic pathways, as summarized in Figure 2 and Tables 1–4; the effects of these drugs on lipid metabolism are described below.

### Nonsteroidal antiinflammatory drugs

Eicosanoids are drivers of inflammation in AIRDs and are major targets of antiinflammatory therapies, including nonsteroidal antiinflammatory drugs (NSAIDs), which inhibit prostaglandin synthesis through the inhibition of COX enzymes (ref. 20 and Table 1). NSAIDs include both nonselective NSAIDs (e.g., aspirin, ibuprofen, diclofenac, phenylbutazone, mefenamic acid) and selective COX-2 inhibitors (e.g., celecoxib and rofecoxib) (38). The antiinflammatory potency of COX-2 inhibitors is higher than that of nonselective NSAIDs, which is reflected in better clinical outcomes in RA and other types of inflammatory arthritis (39, 40). While all COX inhibitors are effective at treating inflammation, their side effects include cardiovascular, gastrointestinal, and renal complications (41). Of relevance to AIRDs is the association of selective COX-2 inhibition with increased risk of thrombotic events. Thromboxane A_2_ is an eicosanoid lipid mediator derived mainly from activated platelets, which constitutively express only COX-1. Thromboxane A_2_ induces vasoconstriction, endothelial adhesion molecule expression, and platelet aggregation and production among other effects, and is elevated in cardiovascular and inflammatory diseases; whereas COX-2 mediates the production of prostacyclin, which mediates vasodilation, inhibits platelet aggregation, and restrains the cardiovascular effects of thromboxane A_2_. Thus, both thromboxane A_2_ and prostacyclin are important mediators of CVD risk (42). CVD risk may also be associated with localized alterations in prostaglandin metabolism and PPARγ activity where the COX isoforms are coexpressed in atherosclerotic plaques (43, 44). Consequently, while COX-1 inhibition protects against atherosclerotic progression (for example, low-dose aspirin inhibits platelet-derived thromboxane A_2_), selective COX-2 inhibitors block cardioprotective prostacyclin and are associated with increased CVD risk (45). In clinical practice, individual CVD risk can be predicted using validated clinical scores; however, the complexity and heterogeneity of therapeutic responses and their direct or indirect impact on lipid metabolism could influence long-term outcomes (45).

### Disease-modifying antirheumatic drugs

Disease-modifying antirheumatic drugs (DMARDs) inhibit inflammatory immune cell responses through various mechanisms (Figure 2 and Tables 1 and 2) and have been used reliably to treat AIRDs for many years. However, DMARD use can be associated with dyslipidemia, driven either by the impact of drugs on the liver or by the toxic side effects of drug metabolites (46, 47). More recent insights into the lipid metabolic pathways influenced by many DMARDs have provided new understanding of their antiinflammatory and immunomodulatory properties.

#### Prednisolone.

Prednisolone (glucocorticoid steroid hormone) effectively reduces inflammation, but long-term use has many side effects, including hypertension, obesity, dyslipidemia, and atherosclerosis (refs. 48–50 and Table 1). The mechanisms underpinning these effects could be associated with the promotion of fatty acid synthase and acetyl-CoA carboxylase activity, as well as inhibition of fatty acid β-oxidation by blocking of acyl-CoA dehydrogenase activity (ref. 51 and Figure 1D). Together, these processes result in hepatic fat accumulation and increased circulating triglycerides and VLDL. There is evidence that low-dose prednisolone attenuates postprandial suppression of lipid oxidation in patients with RA (52). Prolonged prednisolone use exacerbates dyslipidemia despite the preferential antiinflammatory effects of treatment (53, 54), although some studies show that RA patients treated with prednisolone can have increased levels of HDL (55). Hepatic lipid accumulation induced by prednisolone can impair insulin signaling through increased activation of MAPK signaling (51). As with many therapies, it has been important to measure these adverse metabolic effects against clinical benefits (48). Baseline lipid profile is considered when patients are started on corticosteroids, but the clinical need for treatment likely takes priority. If prednisolone treatment is of short duration (e.g., acute flare of gout, bridging therapy in RA) (56), then dyslipidemia is not clinically managed in any particular way. However, long-term prednisolone treatment requires monitoring of lipid profile as indicated in various guidelines and as part of CVD risk management recommendations (53, 57).

#### Hydroxychloroquine.

Despite the widespread use and efficacy of hydroxychloroquine in the treatment of AIRDs (58), its mechanism of action is relatively unclear. It has beneficial effects on lipid and glucose metabolism but also exerts multiple other immunomodulatory actions (ref. 59 and Table 1). The atheroprotective role of hydroxychloroquine is likely due to both its antiinflammatory and its lipid-modifying effects. Hydroxychloroquine is protective against endothelial cell damage, hypertension, and thrombosis (CVD risk factors) via the inhibition of endothelin-1 (a potent vasoconstrictor), downregulation of proinflammatory cytokines such as TNF-α, and reduced levels of proinflammatory ROS, which inhibit platelet aggregation (60–62). The mechanisms underlying its direct effect on lipid metabolism remain largely unknown. There is evidence that hydroxychloroquine reduces atherogenic triglycerides and total and LDL-cholesterol and increases atheroprotective HDL-cholesterol (63, 64). Hydroxychloroquine is also a substrate for CYP enzymes, which are associated with the metabolism of many drugs; thus, it could interfere with the efficacy of combination therapies that are metabolized via the CYP pathway, including calcineurin inhibitors (59, 65), or increase the cardiovascular risk in association with various antibiotics (66). Hydroxychloroquine-mediated changes in lipid metabolism could influence immune cell function. Interestingly, research investigating hydroxychloroquine in SARS-CoV-2 infection shows that the drug binds to sialic acid–containing glycolipids within plasma membrane lipid rafts and inhibits viral uptake (67). It has also been shown to disrupt lysosomal membranes; therefore, hydroxychloroquine could also mediate its effects in AIRDs by modifying lipid raft–mediated immune cell signaling, which can in turn modulate immune cell function (refs. 9, 68, and Figure 1A).

#### Calcineurin inhibitors.

Calcineurin inhibitors (cyclosporin, voclosporin, tacrolimus) block T cell signaling and activation (Table 1) but also have noteworthy off-target effects, including impairment of endothelial cell function associated with COX-2 inhibition and reduced production of prostaglandin E_2_ (69) and dyslipidemia (increased total cholesterol, LDL-C, triglycerides, and apolipoprotein B) (70, 71). Various mechanisms could contribute to altered lipid levels, including reduced hepatic LDL-C clearance and increased cholesterol biosynthesis via the HMG-CoA pathway mediated by inhibition of 27-hydroxycholesterol, an oxysterol that inhibits cholesterol metabolism via HMG-CoA (ref. 72 and Figure 1C). Interestingly, voclosporin, recently approved for use in adult lupus nephritis, shows a significant reduction in total cholesterol and LDL-C, potentially due to its superior antiinflammatory properties (73). Cyclosporin also inhibits bile acid synthesis via 26-hydroxylase and could reduce triglyceride degradation by inhibiting lipoprotein lipase activity (71, 74). Thus, although calcineurin inhibitors are favorable in AIRDs, further mechanistic research is required to assess the antiinflammatory benefits against the off-target effects of blocking fundamental metabolic processes.

#### Mycophenolate mofetil and azathioprine.

Mycophenolate mofetil (MMF) and azathioprine inhibit cellular proliferation through inhibition of purine nucleotide synthesis pathways (ref. 75 and Table 2). Mycophenolic acid (the active metabolite of MMF) can also activate PPARγ (76) and increase intracellular lipids including fatty acids, cholesterol, and phosphatidylcholine in vitro (77). Such metabolic dysregulation could contribute to MMF function via disruption of cell signaling and membrane integrity. Another study shows that azathioprine reduced abnormally upregulated cellular cholesterol/lipid biosynthesis and uptake and induced ER stress and apoptosis in glioblastoma; this effect was likely mediated by blocking of EGFR/AKT/SREBP-1 signaling and not via the typical ABCA1-mediated cholesterol efflux through the LXR transcription factor, as neither LXR nor ABCA1 levels were altered by azathioprine (78). Interestingly, small-molecule inhibitors of sterol regulatory element–binding protein (SREBP) such as betulin, in addition to their antitumoral effects (79), can reduce cholesterol and fatty acid biosynthesis and atherogenic hyperlipidemia in animal models, suggesting that azathioprine could have a similar effect (80). SREBP-1 also reduces proinflammatory signaling and modulates macrophage phagocytosis (81, 82), additional pathways that could be affected by the inhibition of this transcription factor.

#### Methotrexate, sulfasalazine, and leflunomide.

Methotrexate suppresses lymphocyte proliferation and cytokine production and increases apoptosis via multiple metabolic pathways (Table 2). Patients with RA have atypically reduced lipid levels considering their increased CVD risk (14); in line with this, recent studies show that methotrexate increases total cholesterol and LDL while reducing CVD risk (83), potentially by restoring normal lipoprotein metabolism (84, 85), although reduced proinflammatory cytokine levels and associated inflammation are also likely to play a role (86). The antiinflammatory mechanisms of sulfasalazine are also thought to have cardioprotective effects (87), potentially mediated by scavenging of oxygen radicals leading to decreased lipid peroxidation, inhibition of arachidonic acid metabolism via COX enzymes that results in reduced platelet aggregation, and inhibition of NF-κB signaling (88–91). Alternatively, sulfasalazine can induce ferroptosis, although it is not established whether this influences drug efficacy (ref. 36 and Figure 1F). Leflunomide, another antiproliferative drug, is known to increase hypertension and thus increase CVD risk, although the mechanism is unknown (ref. 92 and Table 2).

#### Cyclophosphamide.

Cyclophosphamide can reduce LDL and VLDL levels and increase acetate (a lipid metabolism by-product) in lupus nephritis patients (93). However, acrolein, a cyclophosphamide metabolite (Table 1), can result in dose-related cardiotoxicity, which is a limiting factor for cyclophosphamide use (94). Acrolein alters levels of heart fatty acid–binding proteins, which deplete antioxidants and ATP levels through altered mitochondrial β-oxidation, thereby reducing the cellular energy pool. Together, these metabolic changes increase apoptosis in cardiomyocytes and can lead to heart failure and myocardial infarction (94). These off-target metabolic effects require close cyclophosphamide dose monitoring and modification in patients with AIRDs. There are few other reports that cyclophosphamide influences metabolite levels in AIRDs (95).

## Target synthetic DMARDs

Target synthetic DMARDs (tsDMARDs) are small-molecule inhibitors used increasingly to treat AIRDs since they are less toxic, have fewer adverse effects, and have increased specificity to proteins and signaling pathways associated with disease pathogenesis (96). An array of tsDMARDs exist targeting key proinflammatory signaling pathways that are stimulated by inflammatory mediators (cytokines, chemokines, growth factors, and antigens), including JAK, MAPK, NF-κB, and spleen-associated tyrosine kinase (SYK)/Bruton’s tyrosine kinase (BTK) pathways (refs. 96–98 and Table 3). The full impact of inhibition of these pathways on specific metabolic mechanisms is unclear but likely plays an important role in the performance of specific tsDMARDs. Furthermore, crosstalk between various signaling pathways adds complexity to therapeutic strategies; for example, NF-κB target genes can inhibit MAPK signaling (99).

### JAK inhibitors

JAK inhibitors block cell signaling via the JAK/STAT pathway (Table 3) but also have cell metabolic effects (including decreased mitochondrial membrane potential, mitochondrial mass, and ROS and inhibition of metabolic genes in synovial tissue) (100) and modify systemic lipid metabolism. Tofacitinib and baricitinib significantly increased HDL-C and LDL-C compared with baseline and other DMARD treatments alone in randomized controlled trials in RA and SLE (101–106), an effect reversed by statins (107). JAK inhibitors also improve HDL function by increasing the activity of lecithin-cholesterol acyltransferase (LCAT; an enzyme that converts free cholesterol to cholesterol esters and supports cholesterol efflux to lipoproteins), thereby increasing HDL efflux capacity (refs. 103, 106, and Figure 1C). Other effects such as alterations in lipoprotein size and content have been described (103, 108); therefore, these therapies may contribute to drug-induced dyslipidemia and exacerbate the lipid imbalances already associated with AIRDs. Past trials have highlighted concerns surrounding the risk of arterial and venous thrombotic events with JAK inhibition, and emerging evidence suggests that this risk is dependent on JAK selectivity and is potentially confounded by indication (109, 110). Based on a review of a randomized controlled trial of tofacitinib versus anti-TNF treatment, the Food and Drug Administration issued an urgent revision for all JAK inhibitors to include information about potential increased risks of serious heart-related events, cancer, blood clots, and death. These emerging concerns are mirrored in recommendations to assess the benefits and risks for patients before initiating or continuing JAK inhibitor therapy (111).

### Targeting the MAPK pathway

The MAPK pathway, comprising ERK, JNK, and p38 kinase (p38) (112), regulates cellular function via activation of transcription factors (Table 3). Although targeting of MAPKs such as p38 by VX-702 has shown clinical benefit in RA and animal models of SLE, the use of MAPK inhibitors is confounded by the vast and pleiotropic effects of MAPKs on immune cell functions and cellular metabolism; this has resulted in multiple failures of MAPK inhibitors in clinical trials (96, 113). Mouse models of kinase deficiency clearly show that interconnected metabolic relationships exist between kinase function and liver-mediated lipid metabolism; altered activity and expression of MAPKs and their inactivating phosphatases are present in models of metabolic disease (114). Notably, downstream insulin signaling stimulates MAPK signaling, ERK can phosphorylate SREBP-2 (a regulator of cholesterol biosynthesis) (115), and ERK/JNK phosphorylates PPARγ (116), linking the MAPK pathway to key regulators of lipid metabolism. The MAPK pathway can also be activated by JAK/STAT signaling (117).

### Targeting NF-κB signaling

Aberrant NF-κB signaling is implicated in many inflammatory (RA, SLE) and metabolic (atherosclerosis, obesity, diabetes) diseases (118, 119). Iguratimod, an inhibitor of RelA, a component of the NF-κB heterodimer, is approved for use in patients with RA in China and Japan (refs. 120, 121, and Table 3). Iguratimod could also affect the cellular metabolic responses associated with NF-κB signaling, including macrophage foam cell formation (lipid accumulation), via reduced expression of lipid transporters (ABCA1 and ABCG1), reduced cholesterol efflux, and increased lipid uptake via scavenger receptors (refs. 122, 123, and Figure 1C). Blocking NF-κB signaling could therefore increase cellular cholesterol efflux and reduce lipid uptake, with both atheroprotective implications through reduced foam cell formation (124) and antiinflammatory benefits via modulation of cell plasma membrane lipid rafts and reduction of Toll-like receptor trafficking and signaling (125). Alternatively, inhibition of NF-κB activation in macrophages can increase atherosclerosis in LDLR-deficient mice (126); disparity between studies may be due to different models and experimental approaches, indicating that such off-target effects need to be studied in more detail in patients. Additionally, the atheroprotective transcription factor PPARα, activated by endogenous fatty acid ligands such as arachidonic acid, can suppress entry of NF-κB to the nucleus owing to increased IκBα expression (127), demonstrating the complexity of NF-κB signaling in lipid metabolism.

### Targeting SYK/BTK pathways

Finally, SYK/BTK–mediated signaling is proximal to multiple downstream signaling pathways, including the MAPK and NF-κB pathways (ref. 128 and Table 3); BTK inhibition can inhibit free fatty acid metabolism in chronic lymphocytic leukemia through reduction of lipoprotein lipase (129). Furthermore, crosstalk between BTK signaling and bioenergetic stress responses in leukemic B cells suggests that cellular metabolic rewiring could mediate the metabolic effects of these molecules (130). Multiple BTK inhibitors are currently in clinical trials for AIRDs.

## Biologics

Biologic therapies are target specific and, despite their eventual catabolism by proteases and complications surrounding anti-drug antibodies, they do not undergo in vivo metabolism within immune cells to elicit their desired effects (ref. 131 and Table 4). Notably, several biologics influence lipid and lipoprotein metabolism, and it is likely that biologics have additional indirect metabolic actions associated with preventing inflammatory cytokines from binding their receptors, as seen with JAK inhibitors (132, 133).

### IL-6 receptor antagonists

Tocilizumab and sarilumab inhibit IL-6 receptor binding to IL-6 and block downstream signaling via multiple pathways, including JAK/STAT, p38/JNK, and MAPK (134). Patients with RA are typically characterized by low levels of LDL, potentially due to LDL-C hypercatabolism driven by the IL-6/acute-phase response (85). By blocking hepatic IL-6 signaling, tocilizumab restores LDL-C metabolism and increases serum LDL-C (85, 135, 136). Tocilizumab also increases HDL and triglyceride serum levels in RA patients (137) and promotes HDL cellular cholesterol efflux capacity (ref. 138 and Figure 1C).

### TNF inhibitors

Treatment with TNF inhibitors (e.g., etanercept or adalimumab) also increases HDL, total cholesterol, and triglycerides, while the apolipoprotein B/apolipoprotein A1 ratio is decreased and LDL-C levels remain unchanged (139). These effects could reduce CVD risk in RA patients (140), potentially by altering the HDL-associated proteome and improving HDL function, when inflammation is reduced by either adalimumab or abatacept (CTLA-4 fusion protein blocking CD80/CD86 costimulation) (141). Interestingly, adalimumab was associated with higher HDL-associated serotransferrin and immunoglobulin J chain and lower serum amyloid A-I in comparison with patients treated with abatacept (141). It has also been shown that RA patients receiving tocilizumab have a greater increase in LDL-C levels compared with those treated with adalimumab (142), highlighting the differential effects of various biologics on lipid metabolism.

### Rituximab

Several studies have reported altered lipid profiles following rituximab (anti-CD20 monoclonal antibody) treatment in AIRDs. In SLE, rituximab reduced triglycerides and resultant atherogenic index of plasma values, likely associated with improvement in disease activity, although reductions in total cholesterol and LDL-C did not reach statistical significance and HDL levels remained stable (143). In contrast, a separate study showed that RA patients treated with rituximab had reduced total cholesterol and HDL levels associated with improved endothelial function and decreased carotid intima-media thickness (144), supporting beneficial metabolic effects. However, another study investigating RA patients responding to rituximab therapy only partially replicated this, showing an increase in total cholesterol and HDL with a paradoxical decreased atherogenic index of plasma and carotid intima-media thickness (145). The disparities between these studies could be dependent on the level of baseline dyslipidemia.

It is plausible that biologic therapies influence systemic lipid metabolism partly via the general dampening of inflammation, particularly considering that the liver is largely responsible for circulating lipoprotein metabolism, as seen in transplant recipients (146). This could also be due to altered hepatic cytokine signaling, as, for example, TNF-α can reduce lipoprotein lipase activity and liver metabolism (147), while in hepatic steatosis IL-1 signaling increased fatty acid synthase expression and triglyceride accumulation (148). Alternatively, in RA, blocking hepatic IL-6 signaling (tocilizumab) restored normal LDL catabolism induced by IL-6 suppression of CYP enzymes. Normalizing CYP enzyme expression could also have a wider effect on cell metabolism generally (85). The effect of anti–IL-17 antibodies (secukinumab) on lipid metabolism remains uncertain, with reports showing increased, unchanged, or reduced HDL and cholesterol levels as well as increased triglyceride levels (149). This uncertainty exists even though IL-17, a proinflammatory cytokine implicated in AIRD and atherosclerosis pathogenesis, is known to affect cholesterol and lipoprotein metabolism (150, 151) and promote foamy macrophage formation (152).

Immune cell lipid metabolism could also be influenced by biologics. Recently, IFNs were shown to have differential effects on membrane cholesterol metabolism in macrophages, including downregulation of cholesterol biosynthesis (153, 154); this effect was not observed with TNF-α, IL-1β, or IL-6, suggesting that new therapies such as anifrolumab (anti–type I IFN receptor antibody) could have effects on both systemic (hepatic) and local (immune cell) lipid metabolism. Changes in immune cell lipid metabolism can also influence cell signaling via changes in lipid rafts (9, 68). By binding membrane CD20, rituximab induces its translocation to lipid rafts, which is crucial, under some conditions, for induction of B cell apoptosis and can be prevented by disruption of lipid rafts by cholesterol depletion (155). However, binding of anti-CD20 antibodies can also trigger antiapoptotic signaling via SYK and AKT pathways, an effect that was also inhibited by cholesterol depletion (156, 157). Thus, modulation of lipid rafts, potentially by alteration of lipoprotein-mediated cholesterol uptake or efflux, could influence drug efficacy. Experimental evidence in cancer immunotherapy shows that inhibition of acetyl-CoA acetyltransferase-1 (ACAT1), an enzyme that increases intracellular esterified cholesterol levels, improves the efficacy of anti–PD-1 therapy in melanoma (158). Reduced cholesterol esterification in CD8^+^ T cells increased plasma membrane cholesterol levels and subsequent lipid raft–associated T cell receptor clustering and signaling, thereby increasing T cell cytotoxicity against melanoma growth. ACAT inhibition can also boost the antiviral activity of CD8^+^ T cells against hepatitis B by promoting lipid raft signaling in vitro (159).

## Advances supporting metabolism- and inflammation-targeted therapies in AIRDs

Chronic inflammation and dyslipidemia (which can be exacerbated by current therapies) both contribute to increased CVD risk in patients with AIRDs. However, studies show that lipid-lowering drugs (such as statins) are not sufficient to reduce CVD risk in some AIRDs, possibly because they cannot completely restore the antiinflammatory properties of HDL (160, 161). Thus, an unmet clinical need exists for better therapies to address both inflammation and atherosclerosis.

Altered lipid metabolism is frequently associated with the use of nonselective and targeted AIRD treatments. The impact of therapy on lipid profiles can be beneficial, as in the case of hydroxychloroquine, which reduces LDL-C in SLE (63), or lead to new drug-induced dyslipidemia or exacerbate current dyslipidemia associated with AIRD (Tables 1–4) with various clinical outcomes. In the context of high mortality rates associated with CVD in AIRDs, lipid modification therapies are a key cotherapy of interest. Statins are inhibitors of HMG-CoA reductase, the rate-limiting enzyme in cholesterol biosynthesis, that reduce levels of circulating cholesterol, particularly cholesterol carried in LDL particles. Atorvastatin can reverse tofacitinib-induced elevation of total cholesterol, LDL-C, and triglycerides in patients with RA (107), and patients treated with statins for over 6 months have improved disease activity scores in comparison with conventional RA therapies, supporting a potential beneficial role for statins in patients with active RA (162). Other trials have assessed the use of statins to reduce inflammation. High-dose statins reduced brain atrophy and disability progression in patients with secondary progressive multiple sclerosis in a randomized controlled trial (163, 164). Statins have also been tested in SLE to treat inflammation and dyslipidemia, with mixed outcomes. Some studies show beneficial effects such as improved lipid and inflammatory cytokine levels and reductions in vascular inflammation, atherosclerotic plaque progression, mortality, and morbidity (165–168). However, statins have not met their primary endpoint in clinical trials, including the Atherosclerosis Prevention in Pediatric Lupus Erythematosus (APPLE) trial in children (169) and the Lupus Atherosclerosis Prevention Study (LAPS) in adults (170). Interestingly, although the LAPS 2-year intervention trial did not meet the atherosclerosis primary and secondary endpoints, significant changes in lipid profiles [lipoprotein(a) and total cholesterol] were reported. Difficulties in stratifying patients based on their initial dyslipidemia status as well as their background medication could be the reason for this.

Recent studies of lipoprotein taxonomy in patients with adult and juvenile-onset SLE (171, 172) and multiple sclerosis (173) have highlighted the heterogeneity in patient lipoprotein profiles. Therefore, baseline lipid levels could be important predictors of therapeutic benefit, as has been shown in RA patients treated with tocilizumab and JAK inhibitors, among whom patients with increased lipid levels had a better response to lipid-lowering drugs (107, 135). Other therapies targeting lipid metabolism include reconstituted HDL (shown to reduce plaque in lipid content, macrophage size, and inflammation; ref. 174) and the recently approved statin alternative inclisiran, which increases LDLR levels in the liver (by inhibiting proprotein convertase subtilisin/kexin type 9, the enzyme responsible for LDLR inhibition), thereby reducing LDL-C in the blood by up to 50%, similarly to high-dose statins (175). In the future, new lipid-modifying drugs could be used as an alternative to, or in combination with, statins for patients with AIRDs and dyslipidemia not controlled by conventional treatment and at high risk of cardiovascular events, particularly in those on antiinflammatory treatments that exacerbate dyslipidemia as discussed above.

Some immune receptors that reside in lipid rafts are targeted by AIRD treatments — including CD20 targeted by rituximab (155), CD80/CD86 targeted by abatacept (141), and IL-6R targeted by tocilizumab (176) — suggesting that lipid modification could potentially alter the efficiency of these therapies by regulating membrane turnover of these receptor targets. Some biologic agents require intact lipid rafts to exert their therapeutic function, e.g., rituximab (155–157). Furthermore, pharmacologic inhibition of lipid raft components (cholesterol and glycosphingolipids) using statins and glycolipid synthase inhibitors (*N*-butyldeoxynojirimycin) restored defective lipid raft levels and normalized in vitro function in CD4^+^ T cells from patients with SLE. This included T cell receptor signaling and function and anti-dsDNA antibody production by autologous B cells (10, 177). Interestingly, elevated glycosphingolipid levels in SLE T cells were associated with the increased expression of the LXR master lipid transcriptional regulator, which directly modulates enzymes involved in glycosphingolipid synthesis (9). Whether supplementation with compounds targeting LXR could further modulate lipid rafts and AIRD drug efficacies remains to be explored.

In some circumstances, the dose of lipid-modifying therapies must be adjusted when they are used in combination with AIRD therapies. Tocilizumab normalizes CYP enzyme expression and increases LDL-C; therefore patients on statin cotherapy may require an increased dose to maintain therapeutic lipid-lowering benefits (135). Cyclosporin can also affect the pharmacokinetics of statins through the inhibition of both organic anion transporter polypeptide-1B1 and CYP3A4 (178). Also, lipids including HDL play an important role as S1P chaperones; therefore, alterations in lipoprotein metabolism could influence the efficacy of drugs modulating the S1P pathway (e.g., fingolimod), which are now used in multiple sclerosis and being investigated in AIRDs (34, 179).

Dietary patterns also modify inflammation; those with a higher inflammatory potential are significantly associated with unfavorable lipid profiles and a higher incidence of CVD (180). Despite these observations, the relationship between nutrition and inflammation in AIRDs is not well established. Oral lipid supplements may aid the effectiveness of conventional therapies, such as essential fatty acid supplementation to increase STM levels; these have been linked to decreased joint pain and predict DMARD responsiveness in RA (31). Dietary polyunsaturated fatty acids can also inhibit ferroptosis (181) and incorporate into T cell membranes, thus altering plasma membrane phospholipid expression and the localization of immunogenic receptors such as IL-2 receptor and Fc receptors into lipid raft microdomains (182). Dietary intervention to alter blood lipids can be beneficial in SLE and RA and reduce disease activity scores (183–185). Increased dietary intake of omega-3 fatty acids increased HDL and reduced triglycerides in juvenile-onset SLE (183, 186) and increased HDL and reduced VLDL in adult SLE (187). Thus omega-3 dietary supplements could be promising therapeutic options for some patients. In contrast, a randomized controlled trial of dietary restrictive patterns reduced weight and fatigue in adults with SLE, but did not affect disease activity or cardiovascular parameters including lipid profiles and inflammatory markers (188).

## Conclusion

Understanding how lipid metabolism influences immune responses and the effect of both conventional and new therapies on lipid metabolism is an ongoing challenge but could identify new ways to target AIRDs. Better control of inflammation using optimal combinations of immunosuppressive treatments, as shown in inflammatory bowel disease (189), could lead to an improved metabolic/lipid profile in AIRDs. Improved monitoring of pro-/antiinflammatory lipoprotein fractions using a granular lipoprotein taxonomy approach and improved CVD risk stratification biomarkers (171, 172), rather than total HDL/LDL levels, could improve targeted patient management. This is relevant since statins do not completely normalize proinflammatory HDL fractions (160). Such improved monitoring could enable novel combination interventions, such as nonspecific dietary intervention with specific lipid lowering and targeted antiinflammatory therapy. Finally, the clinical relevance of metabolic/lipid biomarkers in AIRDs needs to be explored in long-term studies to capture the long-term toxicity of combined therapies as well as their impact on cardiovascular events.

## Figures and Tables

**Table 1:**
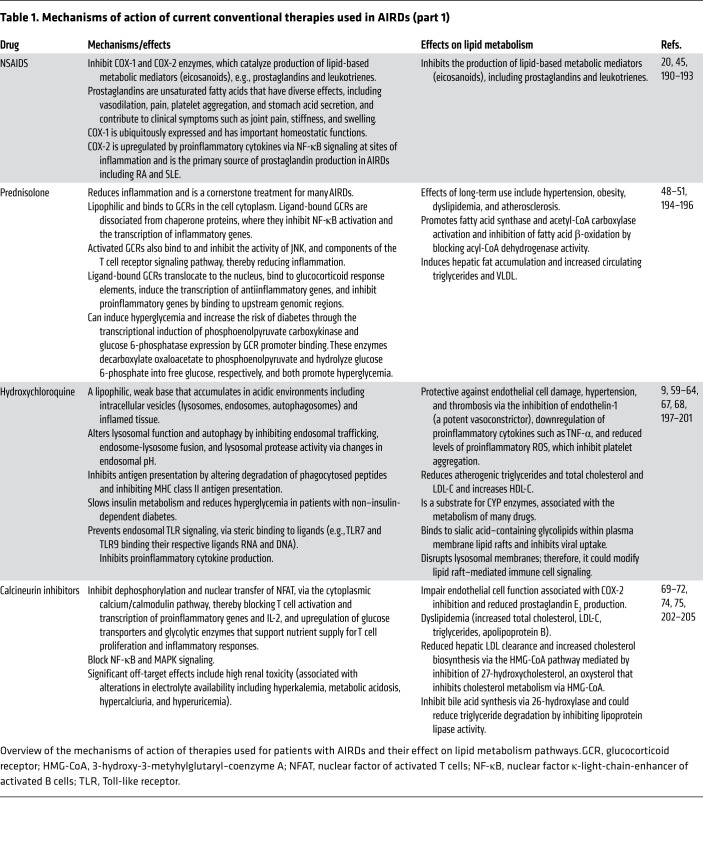
Mechanisms of action of current conventional therapies used in AIRDs (part 1)

**Table 2:**
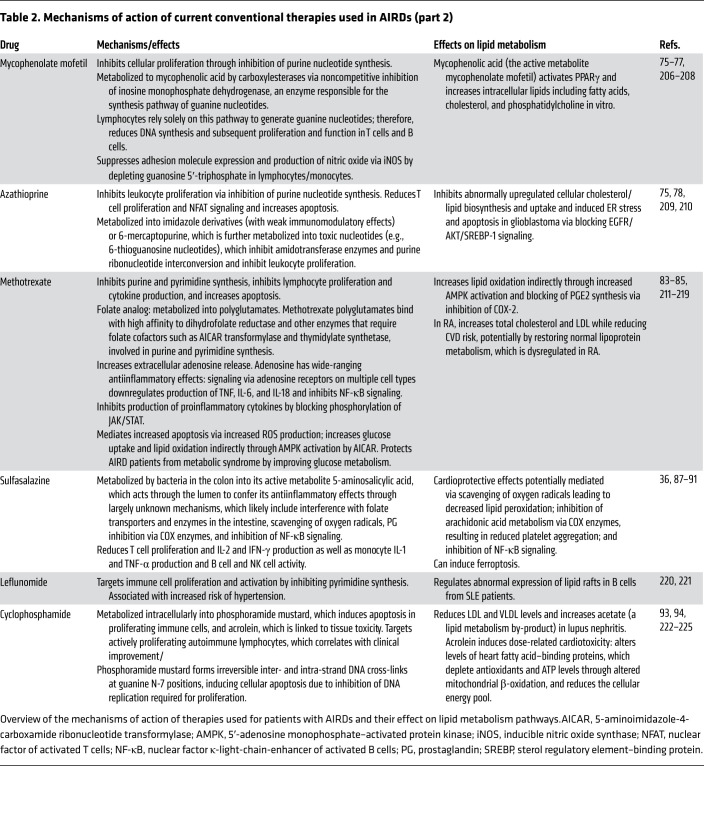
Mechanisms of action of current conventional therapies used in AIRDs (part 2)

**Table 3:**
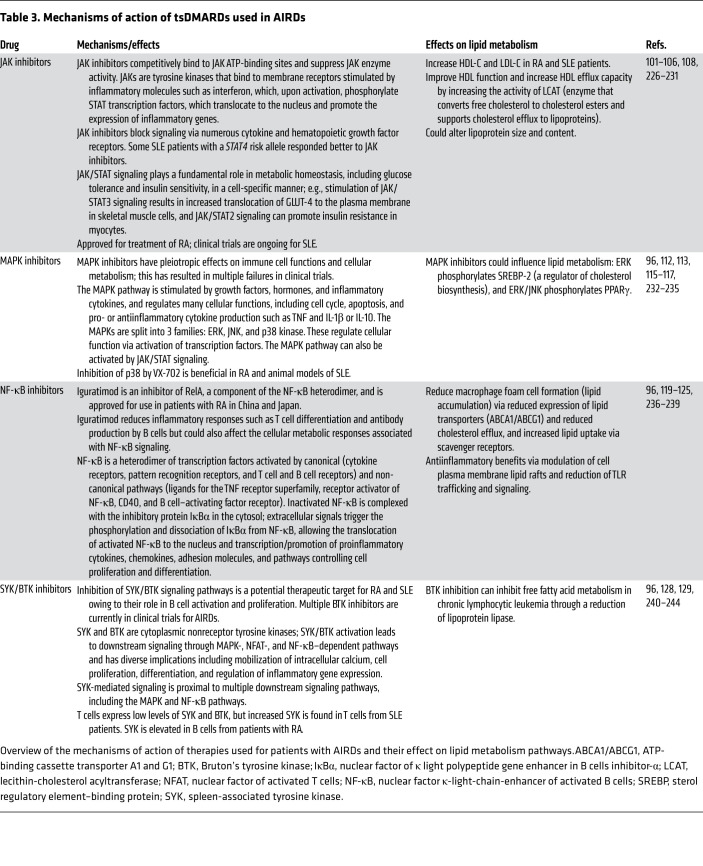
Mechanisms of action of tsDMARDs used in AIRDs

**Table 4:**
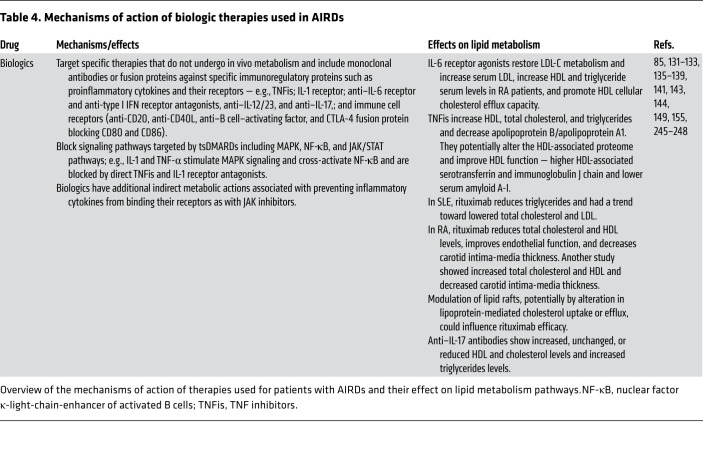
Mechanisms of action of biologic therapies used in AIRDs

## Version 1 01/18/2022

Electronic publication
